# Modulation of chimeric antigen receptor surface expression by a small molecule switch

**DOI:** 10.1186/s12896-019-0537-3

**Published:** 2019-07-03

**Authors:** Alexandre Juillerat, Diane Tkach, Brian W. Busser, Sonal Temburni, Julien Valton, Aymeric Duclert, Laurent Poirot, Stéphane Depil, Philippe Duchateau

**Affiliations:** 1 Cellectis Inc, 430E, 29th street, New York, NY 10016 USA; 2 Cellectis, 8 rue de la croix Jarry, 75013 Paris, France

**Keywords:** Chimeric antigen receptor, Cell engineering, Small molecule switch

## Abstract

**Background:**

Engineered therapeutic cells have attracted a great deal of interest due to their potential applications in treating a wide range of diseases, including cancer and autoimmunity. Chimeric antigen receptor (CAR) T-cells are designed to detect and kill tumor cells that present a specific, predefined antigen. The rapid expansion of targeted antigen beyond CD19, has highlighted new challenges, such as autoactivation and T-cell fratricide, that could impact the capacity to manufacture engineered CAR T-cells. Therefore, the development of strategies to control CAR expression at the surface of T-cells and their functions is under intense investigations.

**Results:**

Here, we report the development and evaluation of an off-switch directly embedded within a CAR construct (SWIFF-CAR). The incorporation of a self-cleaving degradation moiety controlled by a protease/protease inhibitor pair allowed the ex vivo tight and reversible control of the CAR surface presentation and the subsequent CAR-induced signaling and cytolytic functions of the engineered T-cells using the cell permeable Asunaprevir (ASN) small molecule.

**Conclusions:**

The strategy described in this study could, in principle, be broadly adapted to CAR T-cells development to circumvent some of the possible hurdle of CAR T-cell manufacturing. This system essentially creates a CAR T-cell with an integrated functional rheostat.

**Electronic supplementary material:**

The online version of this article (10.1186/s12896-019-0537-3) contains supplementary material, which is available to authorized users.

## Background

In the past few years, the adoptive transfer of engineered T-cells has emerged as a key player in the development of new treatments against cancer [1, 2]. The success of such therapies relies, in part, on the ability to engineer chimeric antigen receptor (CAR) to target tumor cells that present a predefined antigen. Adoptive T-cell therapy with CAR-expressing T-cells targeting the B cell antigen CD19 have induced durable, sustained antitumor responses in patients with leukemias and lymphomas. Inspired by this success, the scientific community has been quickly extended the number and identity of targeted tumor antigen far beyond CD19, raising new challenges in the antigen selection and for the manufacturing of these engineered cells.

Antigen or non-antigen activation (autoactivation) may lead to T-cell differentation toward effector phenotypes and exhaustion or even T-cell elimination through CAR induced T-cell fratricide depending of the antigen or CAR architecture [3, 4]. In the past years, several molecular approaches to control engineered CAR T-cell, in a spatio-temporal and non-lethal manner, have been developed [5–17]. However, only a handful of these approaches would allow the remote control of the engineered T-*cells* ex vivo, in an on- or off-switch manner. Therefore, there is still a need for systems that precisely control CAR T-cell functions ex vivo in order to circumvent some of the difficulties encountered during manufacturing of these engineered cells and to overall expand and improve the possibilities for producing CAR T-cells targeting novel tumor antigens.

## Results

### Design of a protease-based CAR control system

**Fig. 1:**
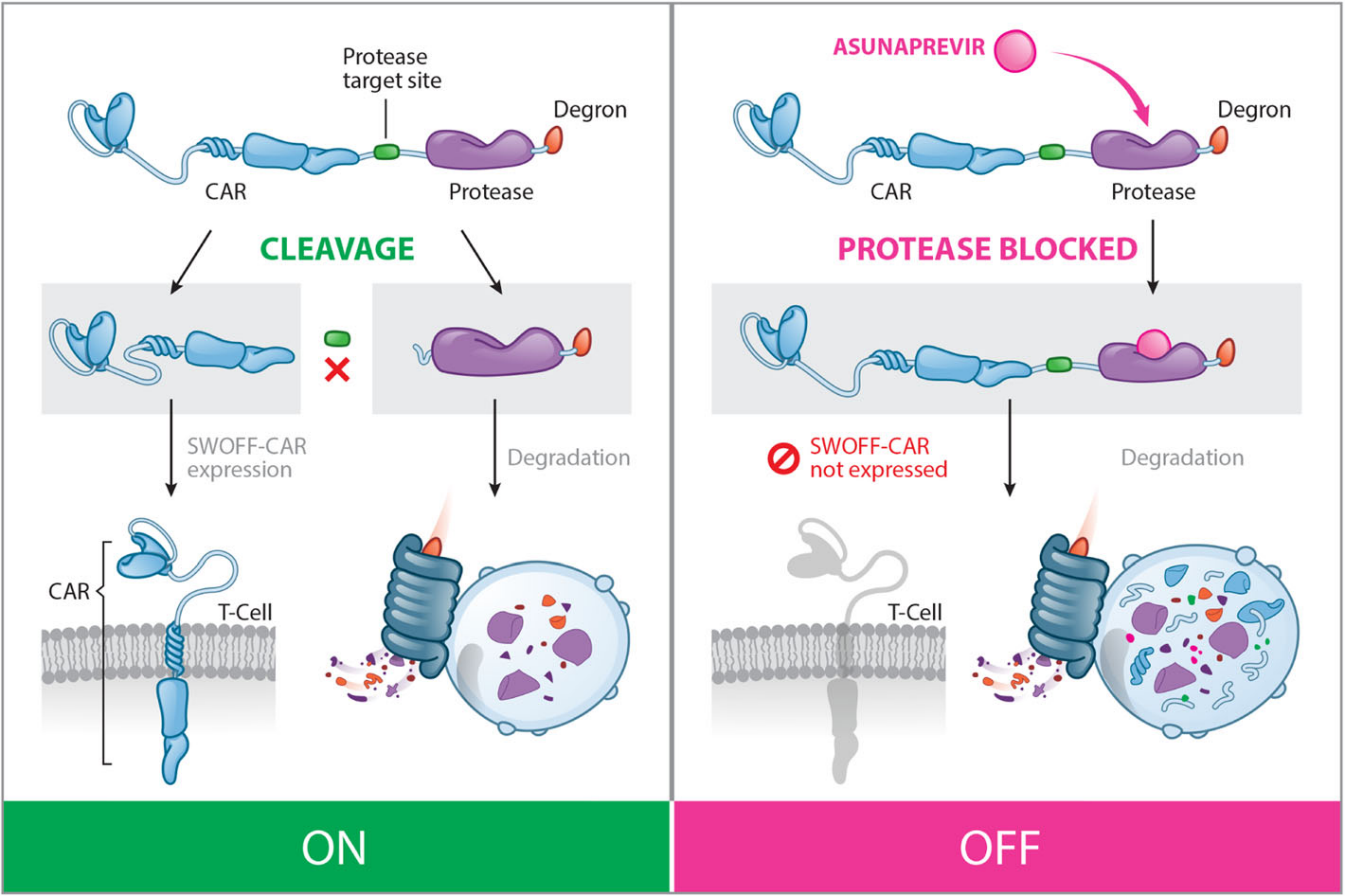
Schematic representation of the SWIFF-CAR principle. The SWIFF-CAR construct is composed of the CAR followed by a protease target site, a protease, and a degradation moiety (degron). In the absence of the protease inhibitor, the degron is cleaved from the CAR, allowing the exposition of the antigen targeting scFV at the T-cell surface (“ON” state, left panel). The presence of Asunaprevir inhibits the cleavage of the degron from the CAR by the HCV NS3 protease, leading to the degradation of the CAR by the T-cell proteolytic pathways (“OFF” state, right panel). Reproduced with permission from Cellectis Group

We sought to control CAR T-cell functions by modulating the presence of the CAR at the cell surface. To do so, we focused on a strategy that would allow us to control the stability and degradation properties of the CAR at the protein level using a small molecule. Recently, Lin and colleagues reported a method that allows the reversible control of protein production using a protease/protease inhibitor [18]. To integrate a protease/protease inhibitor strategy into CAR T-cell technology, we chose the HCV NS3 protease and its inhibitor Asunaprevir (ASN) as an ideal candidate. We therefore modified a second-generation CAR architecture to incorporate the protease/degron component. This component is designed to control the stability of the engineered fusion protein and therefore to modulate CAR surface expression (Fig. 1). We fused the degradation moiety, composed of a protease target site, the HCV NS3 protease, and the degron, to the C-terminal end of the CAR, such that upon cleavage at the protease target site, a short 8-amino-acid sequence would remain at the C-terminus of the CD3z domain that contains the ITAMs (this engineered CAR will be refereed as SWIFF-CAR for switch-off CAR).

**Fig. 2:**
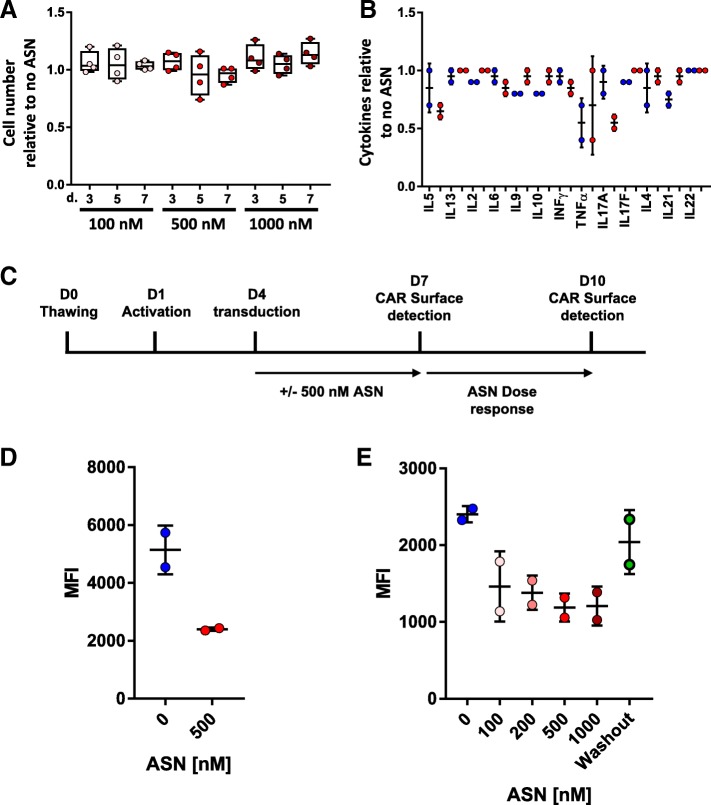
**a** Proliferation of T-cells in the presence of increasing concentrations of Asunaprevir. The total number of cells at different days cultured in presence of 100 nM, 500 nM or 1000 nM relative to 0 nM ASN is presented. Data are shown as the median of PBMC from 2 donors done in duplicate. **b** Cytokine quantification after co-culture of anti-CD22 CAR T-cells with target cells as a function of Asunaprevir concentration. Data are normalized to the maximum value (with or without 500 nM ASN) and shown as the mean ± SD (duplicates). **c** Schematic representation of the experimental setup to determine the effect of ASN on SWIFF-CAR surface expression. **d** MFI of CAR positive cells 3 days post CAR transduction (day 7) in the absence (blue bars) or presence of 500 nM ASN. Data are shown as the mean ± SD (two T-cell donors). **e** MFI of CAR surface detection at day 10 of ASN dose response (0–1000 nM). Blue Dots: No ASN, “Red” dots: dose response of ASN. Green dots: washout of previously ASN (500 nM) treated T-cells. Data are shown as the mean ± SD (two T-cell donors)

The protease inhibitor, ASN, used to control the degradation system is in clinical development, and we hypothesized that it should be functionally inert to T-cells [19–21]. Indeed, Grasela and colleagues reported a dose escalation of ASN (in single or multiple regimen), with the most severe adverse events being headache or diarrhea in a minority of patients [20]. In addition, the lack of reported susceptibility to infections in this report suggested that T-cells were not affected by the drug. Nevertheless, we first evaluated the effects of ASN on the expansion of activated primary T-cells over a period of 7 days. As anticipated, we did not observe significant effects of the small molecule on the proliferation and viability of the T-cells after treatment with 100 nM to 1 μM ASN (Fig. 2a and Additional file 1: Figure S1).

We then evaluated whether the presence of ASN could impair the secretion of key cytokines by CAR T-cells upon activation with target cells. To this end, we first transduced T-cells using lentiviral particles encoding, as proof of concept, a CAR targeting CD22 [22]. In particular, the CAR was composed of single-chain variable fragment (scFv) targeting CD22 antigen fused to a hinge and transmembrane domain derived from the T-cell surface glycoprotein CD8 alpha chain (CD8a). The intracellular domain was composed of signaling domains from co-stimulatory 4-1BB (CD137) followed by the intracytoplasmic signaling region of the ζ-chain of the CD3–T cell receptor. CAR T-cells presenting the anti-CD22 CAR were then co-cultured overnight with target cells presenting the target CD22 antigen in the presence or absence of several concentrations of ASN (0, 100 nM, 500 nM, or 1000 nM). The collected supernatants were then used to quantify 13 different cytokines. Treatment with ASN did not result in notable variations (increases or decreases) in cytokine production (Fig. 2b and Additional file 1: Figure S2). In all, these results show that ASN has no meaningful effects on T cell function that would preclude its further development as a modulator of CAR T-cell expression.

### SWIFF-CAR (switch-off CAR) surface expression can be tuned with Asunaprevir

Having shown that ASN is largely inert against T-cells and CAR T-cells, we next explored the possibility to trigger the down regulation of the SWIFF-CAR on the T-cell surface with ASN. To deliver the SWIFF-CAR into T-cells, we focused on commonly used lentiviral particles and transduced freshly activated PBMCs. The overall decay kinetic of the protease/protease inhibitor system relies on the half-life of the CAR at the cell surface since the addition of the protease inhibitor will only prevent newly synthetized proteins from reaching the surface. Accordingly, we decided to first monitor CAR surface presentation, measured by mean fluorescence intensity (MFI) and positive cells numbers, 48 h after incubation with 500 nM ASN and a labelled CD22 recombinant protein. Using crescent amounts of lentiviral particles allowed reaching a plateau of ~ 70% of CAR positive cells while the MFI continued to increase over the tested range of lentiviral particles doses. By design, the overall amount of CAR protein present at the surface should decrease in the presence of ASN. Indeed, we observed that the addition of ASN to the culture medium markedly decreased the MFI of the CAR-positive population, while the percentage of CAR-positive cells was only slightly decreased (Additional file 1: Figure S3).

Next, we evaluate the possibility to control and tune the CAR surface expression at different time point and using a dose response of ASN (Fig. 2c). We observed that the addition of ASN to the culture medium, immediately after transduction, markedly decreased the MFI of the CAR-positive population (Fig. 2d). The same, transduced, control cells (not treated with ASN) were incubated with different doses of ASN (0, 100, 200,500 or 1000 nM) and cultured for an additional 72 h. We found that the MFI was decreased in a dose dependent manner (Fig. 2e). Importantly, washing-out the ASN after the first 48 h pre-incubation (washout, green dots) allowed to recover an MFI in range with the no drug scenario (blue dots), confirming the reversibility of the system (Fig. 2e).

### The cytolytic functions of SWIFF-CAR T-cells can be tuned with Asunaprevir

To demonstrate that this protease-based approach can be used to control both the surface expression of the desired CAR protein and the cytolytic properties of the engineered T-cells, we conducted additional experiments using SWIFF-CAR T cells. Freshly activated PBMCs were transduced using lentiviral particles encoding the SWIFF-CAR, expanded for 11 days in the presence of IL2, and frozen. To monitor the control (inhibition) of CAR T-cell cytolytic function by ASN, SWIFF-CAR T-cells were thawed and cultured with 0, 1 nM, 20 nM, 100 nM, 200 nM, or 500 nM ASN in the presence of CD22^+^ target cells expressing luciferase, at a ratio of 3 T-cells per target cell. After an overnight coculture, the luciferase signal was measured, and an equal number of fresh target cells was added to the coculture. The procedure was repeated the following day, resulting in 3 coculture periods, period 1 (0-24 h), period 2 (24-48 h) and period 3 (48-72 h). We used this particular experimental setup, target:effector ratio and multiple co-culture periods, to allow increase in cell killing over the different coculture periods and to further follow “kinetics” of killing efficiencies (measure of the luciferase signal). We first verified that the addition of ASN and/or of untransduced T-cells (not expressing the SWIFF-CAR) was not impacting the viability of the target cells (Additional file 1: Figure S4). Because neither the ASN nor the untransduced T-cells were impacting the viability of the target cells, as indicated by less than 10% difference in luminescence compared to the target cells only, we focused on the luciferase measurements where the CD22+ target cells were coincubated with SWIFF-CAR T-cells.

**Fig. 3:**
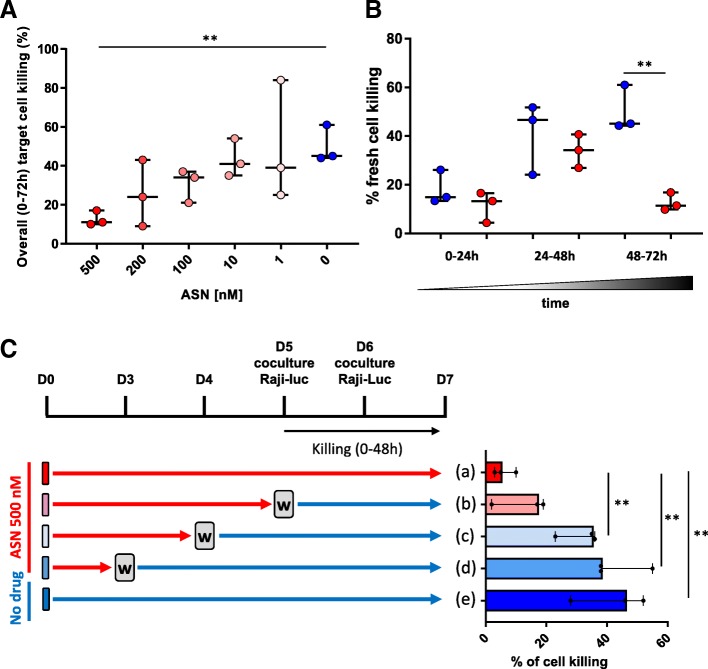
**a** Cytotoxicity (target cell killing) calculated during the 72 h coincubation in function of the ASN concentration (0–500 nM). **b** Cytotoxicity (target cell killing) calculated during the three 24 h periods in presence (500 nM) or absence of ASN. **c** Schematic representation of the washout experiment (top). The grey W boxes indicate the ASN washouts. Cytotoxicity (target cell killing) calculated during the 48 h coincubation. All data are shown as the median with 95% confidence intervals of three independent experiments. *N* = 3. Significance is determined by a standard t-test, * = *p* ≤ 0.05,** = *p* ≤ 0.01

We immediately noticed a clear correlation between target cell survival over the 72 h of coincubation and the amount of ASN (Fig. 3a). We then decipher, for the higher dose of ASN (that results in minimal killing over the 72 h), the target cell killing during the 3 coincubation periods by calculating the proportion of cell killing relative to the amount of freshly added cells (Fig. 3b). By design of the experiment, we observed a low killing efficiency (median: 15%), without influence of the ASN (median: 13%), during the first period of coculture (0-24 h). During the second period (24-48 h), the target cell killing efficiency increased (median:47%) with the premise of an ASN effect on target cell killing (median:34%), although the change was not statistically significant (*p*-value: 0.5, Fig. 3b). However, in coincubation period 3 (48-72 h), we noticed a clear significant correlation between target cell survival and the presence of ASN (median killing: 45% versus 11% with ASN, *p*-value: 0.003. Figure 3b). Altogether, the results we obtained indicated that ASN can tune the surface presentation of the CAR constructs and allow to control the resulting cytolytic properties. These results also clearly indicated that the residual 8-amino-acid sequence that remained at the C-terminus of the CD3z/ITAM CAR domain was not impairing the cytolytic properties of the T-cells.

### The Asunaprevir-based inhibition of cytolytic function is reversible

We next investigated whether the inhibition of the SWIFF-CAR T-cell cytolytic functions using ASN was reversible. We inhibited CAR T-cell cytolytic function by coculturing SWIFF-CAR T-cells with 500 nM ASN. After 3, 4 or 5 days of culture with ASN, we washed-out the ASN and continued culturing the cells (Fig. 3c) to allow surface re-expression of the CAR. These SWIFF-CAR T-cells were then characterized for their ability to kill target cells using the assay described above.

We observed that the washout of the ASN just prior to co-culture (condition (b) in Fig. 3c) with the CD22^+^ target cells increased (2-fold) the killing of the target cells compared to cells cultured in the presence of ASN (condition (a) in Fig. 3c). As expected, the removal and washout of ASN at earlier time points permitted a greater recovery of cytolytic properties. Washing out ASN 24 h prior to the co-culture (condition (c) in Fig. 3c) resulted in a significantly (*p*-value: 0.0056) greater recovery of cytolytic activity than did maintaining SWIFF-CAR T-cells under drug (a 5-fold increase). Washing out ASN 48 h prior to the coculture (condition (d) in Fig. 3c) allowed for a recovery of cytolytic activity equivalent to untreated SWIFF-CAR T-cells (*p*-value: 0.86. Figure 3c).

Altogether, the results presented here make the proof-of-concept that it is possible to reversibly control the cytolytic properties of CAR T-cells using small molecule-dependent tuning of CAR degradation and stability.

## Discussion

CAR T-cell based adoptive immunotherapies are attracting a great deal of attention due to their outstanding success rates [1, 2]. These therapies rely on arming T-cells with chimeric receptors that recognize an antigen specifically expressed on a tumor. Endowing T-cells with a therapeutically relevant CAR may be a challenging process as few truly tumor-specific antigens have been identified. A particularly complicated case arises when the targeted antigen is present not only on the malignant tissue but also on the activated CAR T-cells, which could lead to potential CAR T-cell fratricide (e.g targeting CD5 or CD7 in T-cell malignancies or CD38 in B-cell non-Hodgkin [3, 4, 23]).

With the aim to propose new alternate avenues to some of these possible hurdles encountered during manufacturing and as an alternative to gene editing approaches, we developed a single-component system to control the cytolytic properties of CAR T-cells using a small molecule drug in a switch OFF fashion. Because the density of CAR at the T-cell surface represents a key variable in controlling the cytolytic outcome [24–28], the ability to tune CAR expression levels or stability represents a promising non-lethal strategy for modulating CAR T-cells function. The past few years have seen the development of several systems allowing small molecule-based protein elimination [29–33], including the recently reported single component small molecule–assisted shutoff (SMASh), which acts on newly synthetized protein [18]. The SMASh strategy was based on a method originally developed to visualize newly synthesized proteins and further optimized to control protein production at the post-translational level [18, 34]. We decided to implement such control systems within the CAR construct, allowing to control its stability. By fusing a functional protease/degron moiety to the C-terminus of a CAR and relying on commonly used lentiviral delivery, we were able to generate a functional CAR-T-cell that incorporates a small molecule (ASN protease inhibitor)-dependent switch OFF system.

The overall decay kinetic of such system relies on the half-life of the CAR at the cell surface. The small molecule induced decay and temporal control of surface expression will be influenced by multiple factors. In particular, it has been shown that immune synapses, signaling, and cytotoxic responses by T cells will differ from CARs compared to native TCRs (Davenport), with additional impact of the nature of the CAR costimulatory domain on formation of immunologic synapses [3]. Furthermore, several groups have reported the down-modulation of the CAR surface expression within hours after co-incubation with target cells expressing the CAR antigen [27, 35, 36]. We therefore envisioned that using an ASN-dependent switch OFF system, which is expected to prevent newly synthetized SWIFF-CAR to reach the surface (through degradation), could synergize with the reported target driven down-modulation of the CAR, by maintaining a low level of surface CAR after the first round of CAR engagement.

Here, we made the proof-of-concept that the implementation of a protease-based shut-off system allows to switch OFF the CAR T-cell cytolytic properties within 48 h using the small molecule Asunaprevir, a kinetic in range with other recently described systems [37]. Altogether, the characteristics of the SWIFF-CAR system, kinetics of CAR surface decay, reversibility, small molecule (ASN) inert against T-cells, could make it perfectly suitable as a non-lethal approach to control ex vivo manufacturing of CAR T-cells.

Beyond the presented in vitro proof-of-concept, our ability to translate this technology to in vivo clinical settings remains to be shown. While the clinical management of patients may require an immediate reactivity, as provided by the so-called “suicide gene” systems, however at the cost the termination of the treatment [38–41], switch-off systems with (slower) non-lethal off-kinetics may provide alternative benefits through reversible and progressive control. To explore the in vivo possibilities, detailed preclinical studies will be required to assess fundamental properties such as switch-off triggering, engraftments/proliferation, tumor control and healthy tissue sparing, with the challenge of developing such models.

## Conclusion

We foresee that the constant development of new small molecule-based CAR control approaches will benefit to the clinical application of CAR T-cells, especially by empowering the ex vivo production and/or conditioning of CAR T-cells. Although additional work is required, we anticipate that manufacturing CAR T-cells with the CAR in an off-state (not present at the cell surface) could also decrease or completely eliminate non-specific activation with the benefit to prevent T-cell differentiation, exhaustion or fratricide, overall improving their future in vivo functions. In addition, we hypothesize that the switch-OFF system could be used to promote, in vivo*,* a delayed and gradual increase in the CAR T-cell functions (and their expansion) by ex vivo preswitching-off CAR T-cells. Overall, this could mitigate some of the toxicities that could occur with early intense antitumor responses.

## Methods

### T-cell proliferation

Cryopreserved human PBMCs/T-cells (ALLCELLS, cat # PB006F) were used in accordance with Cellectis IRB/IEC-approved protocols. T-cells were cultured in X-Vivo 15 (Lonza) supplemented with 5% human serum hAB (Gemini) and 20 ng/ml IL-2 (Miltenyi) at a density of 1 × 10^6^ cells/ml.

### Cytokine profiling

T-cells were cocultured with Raji target cells in 12-well culture plates in the presence of various concentrations of ASN for 24 h. Cells were spun down, and the supernatants were aliquoted and frozen. Cytokine levels in the supernatants were measured with LEGEND plex Human Th Cytokine panel (Biolegend).

### Lentiviral particle production

Lentiviral particles were generated in 293FT cells (ThermoFisher) cultured in RPMI 1640 Medium (ThermoFisher) supplemented with 10% FBS (Gibco), 1% HEPES (Gibco), 1% L-Glutamine (Gibco) and 1% Penicilin/Streptomycin (Gibco) using Opti-MEM medium (Gibco) and Lipofectamine 2000 (ThermoFisher) according to standard transfection procedures. 48 and/or 72 h post transfection the supernatants were recovered and concentrated by ultracentrifugation.

### Lentiviral particle T-cell transduction

Human PBMCs () were thawed and plated at 1 × 10^6^ cells/ml in X-vivo-15 media (Lonza) supplemented with 5% hAB serum (Gemini) or CTS Immune Cell SR (ThermoFisher) and 20 ng/ml IL-2 (Miltenyi Biotech) for overnight culture at 37 °C. The next day, the PBMCs were activated using human T activator CD3/CD28 (Life Technology) in serum-free X-vivo-15 media without IL-2. One million activated PBMCs (in 600 μl) were immediately incubated without removing the beads in an untreated 12-well plate pre-coated with 30 μg/ml retronectine (Takara) in the presence of lentiviral particles encoding the engineered SWIFF-CAR for 2 h at 37 °C. Six hundred microliters of 2x X-vivo-15 media (X-vivo-15, 10% hAB serum and 40 ng/ml IL-2) was added after 2 to 3 h, and the cells were incubated at 37 °C for 72 h. If required, transduced T-cells were then expanded for 11 days in G-Rex10 (Wilson Wolf) in 40 ml of complete X-vivo-15 media.

### SWIFF-CAR surface detection

Three to five days after transduction, T-cells were incubated with or without 500 nM Asunaprevir for 48 h. The CAR surface expression was then quantified, by flow cytometry (MACSQuant Analyzer 10, Miltenyi Biotec) using labeled recombinant protein targeted by the CAR (LakePharma) and PE-conjugated goat anti-mouse IgG Fcy (Jackson Immunoresearch).

### Assessment of the SWIFF-CAR cytotoxicity

Transduced T-cells (1.5 × 10^6^ cells) were incubated in X-vivo-15 media with 5% hAB serum, lacking Il-2 supplemented with or without 1 to 500 nM Asunaprevir (Apexbio Technology or MedChem Express) in a 3:1 (T-cells: Targets) ratio with target cells (Raji) presenting the CAR target antigen and expressing a luciferase (0.5 × 10^6^ cells) in a 12-well plate. After 24 h, the cells are collected and mixed, and 100 ul of cells was used for luciferase quantification (OneGlo, Promega). The remainder of the cells were pelleted and resuspended in fresh X-vivo 15 media with 5% hAB serum, no Il-2 (supplemented with or without 1–500 nM Asunaprevir), and an additional 0.5 × 10^6^ target cells were added. This step was repeated for 3 consecutive days.

### Assessment of the ASN wash-out on SWIFF-CAR cytotoxicity

T-cells transduced (lentiviral particles) to express the engineered SWIFF-CAR were incubated in complete X-vivo-15 media supplemented with or without 500 nM of Asunaprevir (Apexbio Technology or MedChem Express). After 72 h a fraction of the cells incubated initially with 500 nM of Asunaprevir were washed and incubated at 37 °C in complete X-vivo-15 (X-vivo-15, 5% hAB serum and 20 ng/ml IL-2) media (corresponding to the wash-out 48 h prior to cytotoxicity assay point). After 96 h, another fraction of the cells incubated initially with 500 nM of Asunaprevir was washed and incubated at 37 °C in complete X-vivo-15 media (corresponding to the wash-out 24 h prior to the cytotoxicity assay point). After 120 h, another fraction of the cells, initially incubated with 500 nM of Asunaprevir, was washed and incubated at 37 °C in complete X-vivo-15 media (corresponding to the wash-out at cytotoxicity assay point). A fraction of the cells was maintained in media containing 500 nM of Asunaprevir (corresponding to the no wash-out point).

The different fractions of transduced T-cells were incubated in X-vivo-15 media supplemented with 5% hAB serum, lacking IL-2 (the no-wash-out point) or washout for all other points with 500 nM of Asunaprevir (Apexbio Technology or MedChem Express) in a 3:1 ratio with target cells (Raji) presenting the CAR target antigen and expressing a luciferase in 12-well plates. After 24 h, the cells were mixed, and 100 μl was used for luciferase quantification (0-24 h period) (OneGlo, Promega). The remainder of the cells were spun down, and the media were replaced with fresh media and additional Raji cells for the 24-48 h period.

### Statistical analysis

The statistical analyses were conducted with GraphPad Prism Software Version 7.04 (GraphPad Software Inc). Significant differences between groups were determined by standard t-tests.

## Additional file

Additional file 1: **Figure S1.** Proliferation of T-cells in the presence of increasing concentrations of Asunaprevir. The total number of cells at different days cultured in presence of 100 nM, 500 nM or 1000 nM relative to 0 nM ASN is presented. (A) Donor 1. (B) Donor 2. The mean value +/− s.d. of duplicates is presented. **Figure S2.** Cytokine quantification after co-culture of anti-CD22 CAR T-cells with target cells as a function of Asunaprevir concentration. Data are shown as the mean ± SD of duplicates per points. **Figure S3.** Dose response transduction of primary T-cells with anti CD22 SWIFF-CAR in the absence (blue bars) or presence of 500 nM Asunaprevir (red bars, two different providers). (A) Percentage of CAR-positive cells. (B) MFI of CAR positive cells. A representative example from 2 experiments is displayed. **Figure S4.** Luciferase signal measured at the different time points (the signal is normalized to the highest value of each experiments) for cocultures with: no T-cells (green), untransduced T-cells (green). Data are shown as the median with 95% confidence intervals of three independent experiments. *N* = 3. (DOCX 399 kb)

## Data Availability

The datasets used and/or analyzed during this study are available from the corresponding authors upon reasonable request.

## Abbreviations

ASN: Asunaprevir
CAR: Chimeric antigen receptor
ITAM: Immunoreceptor tyrosine-based activation motif
MFI: Mean fluorescence intensity
PBMC: Peripheral blood mononuclear cell
SMASh: Small molecule–assisted shutoff
SWIFF-CAR: Switch-off CAR
TCR: T-cell receptor
