# Supplementary material for: Modulation of chimeric antigen receptor surface expression by a small molecule switch
Source: BMC Biotechnol. 2019 Jul 3;19:44. doi: 10.1186/s12896-019-0537-3 (PMC6610870; doi:10.1186/s12896-019-0537-3)
Supplement: Supplementary file 1 — Figure S1. Proliferation of T-cells in the presence of increasing concentrations of Asunaprevir. The total number of cells at different days cultured in presence of 100 nM, 500 nM or 1000 nM relative to 0 nM ASN is presented. (A) Donor 1. (B) Donor 2. The mean value +/− s.d. of duplicates is presented. Figure S2. Cytokine quantification after co-culture of anti-CD22 CAR T-cells with target cells as a function of Asunaprevir concentration. Data are shown as the mean ± SD of duplicates per points. Figure S3. Dose response transduction of primary T-cells with anti CD22 SWIFF-CAR in the absence (blue bars) or presence of 500 nM Asunaprevir (red bars, two different providers). (A) Percentage of CAR-positive cells. (B) MFI of CAR positive cells. A representative example from 2 experiments is displayed. Figure S4. Luciferase signal measured at the different time points (the signal is normalized to the highest value of each experiments) for cocultures with: no T-cells (green), untransduced T-cells (green). Data are shown as the median with 95% confidence intervals of three independent experiments. N = 3. (DOCX 399 kb) [file 12896_2019_537_MOESM1_ESM.docx]

**Additional file 1**

**
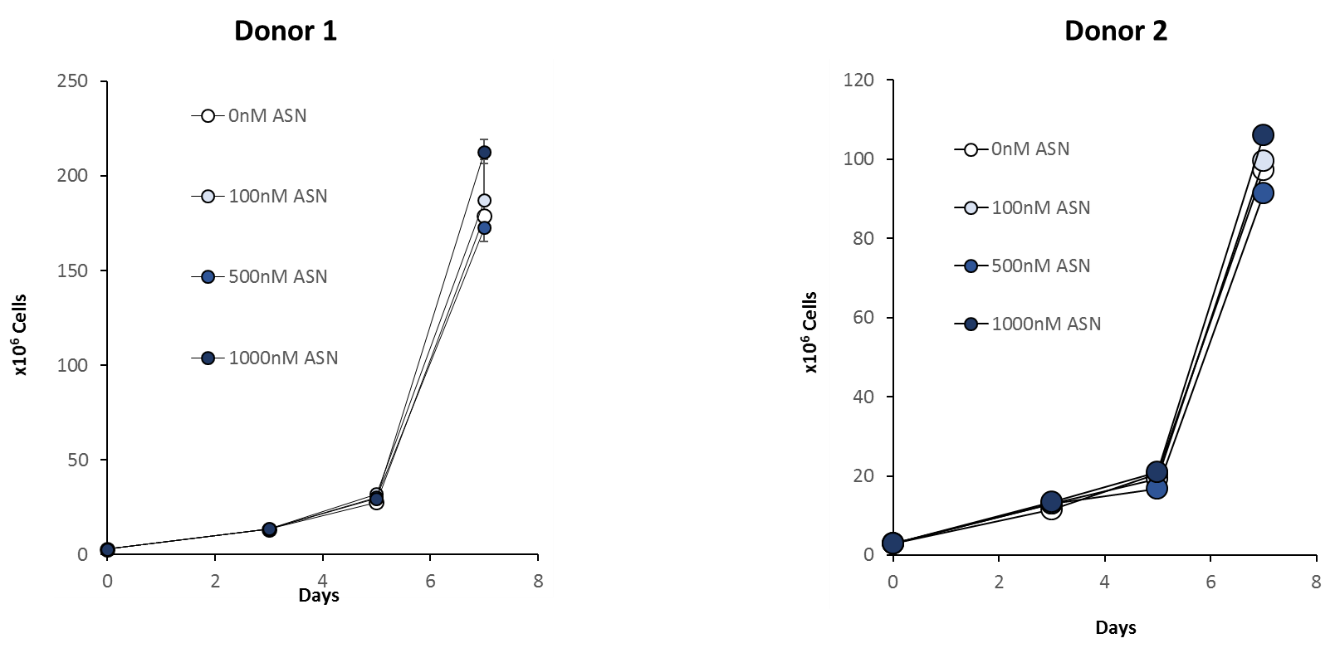
**

**Figure S1:** Proliferation of T-cells in the presence of increasing concentrations of Asunaprevir. The total number of cells at different days cultured in presence of 100 nM, 500nM or 1000 nM relative to 0 nM ASN is presented. (A) Donor 1. (B) Donor 2. The mean value +/- s.d. of duplicates is presented.

**
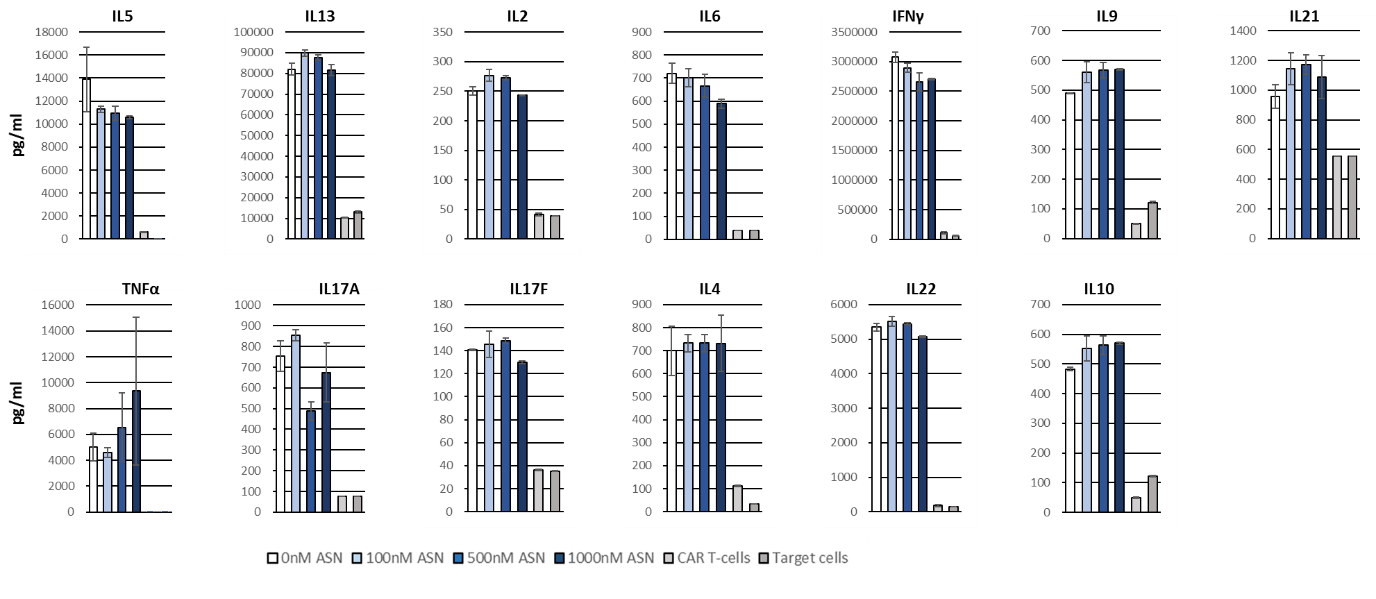
**

**Figure S2:** Cytokine quantification after co-culture of anti-CD22 CAR T-cells with target cells as a function of Asunaprevir concentration. Data are shown as the mean ± SD of duplicates per points.

**
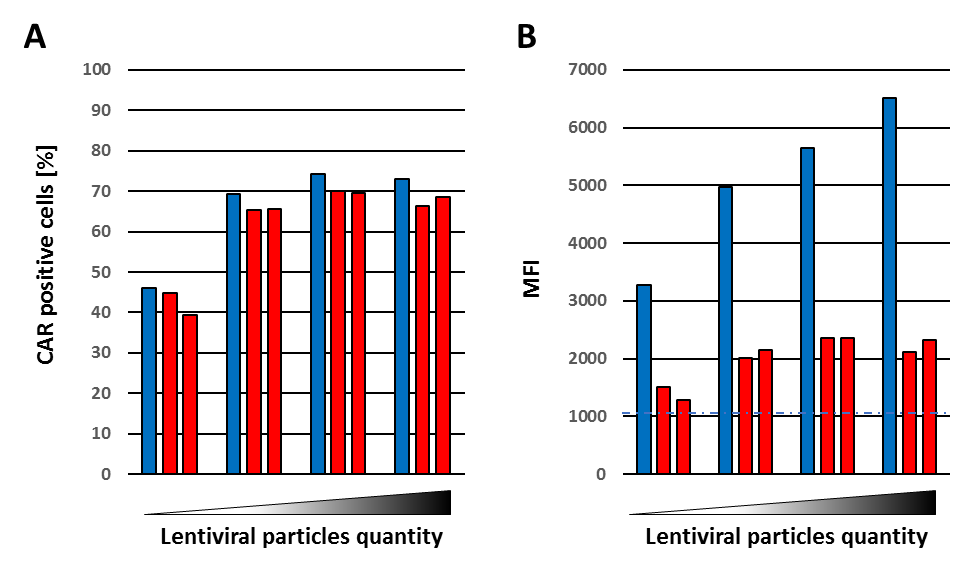
**

**Figure S3:** Dose response transduction of primary T-cells with anti CD22 SWIFF-CAR in the absence (blue bars) or presence of 500nM Asunaprevir (red bars, two different providers). (A) Percentage of CAR-positive cells. (B) MFI of CAR positive cells. A representative example from 2 experiments is displayed.

**
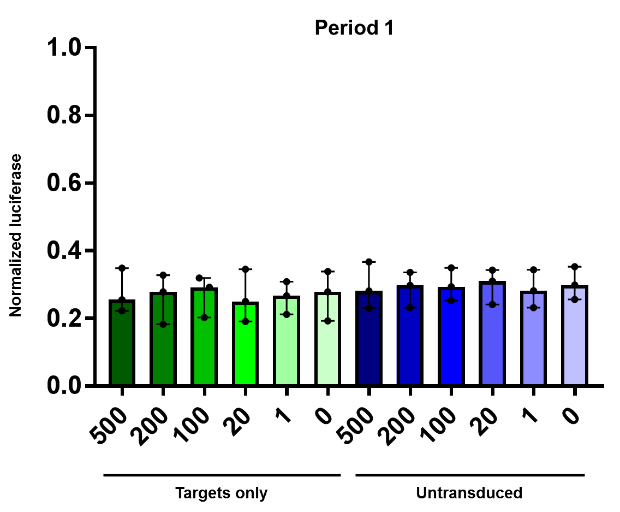

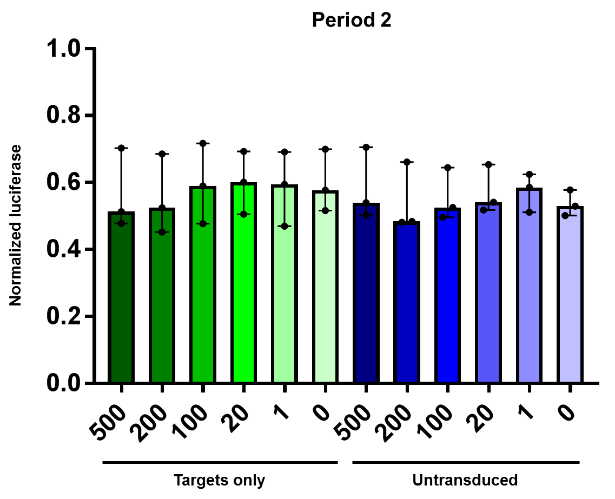
**

**
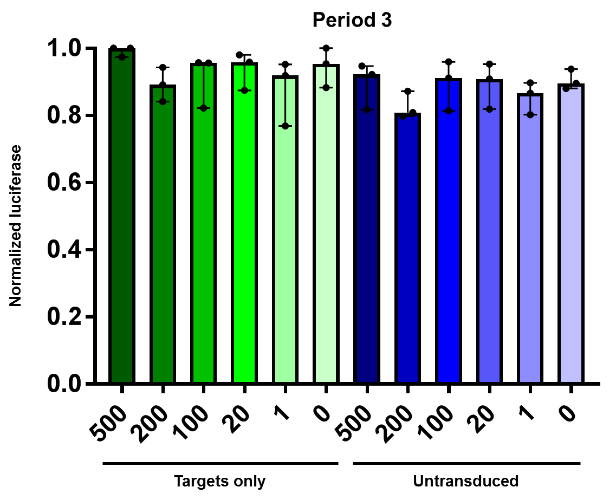
**

**Figure S4:** Luciferase signal measured at the different time points (the signal is normalized to the highest value of each experiments) for cocultures with: no T-cells (green), untransduced T-cells (green). Data are shown as the median with 95% confidence intervals of three independent experiments. N=3.
